# Long-term consequences of early marriage and maternity in West and Central Africa: Wealth, education, and fertility

**DOI:** 10.7189/jogh.11.13004

**Published:** 2021-08-10

**Authors:** Vera Sagalova, Siméon Nanama, Noel Marie Zagre, Sebastian Vollmer

**Affiliations:** 1 Heidelberg Institute of Global Health, University of Heidelberg, Heidelberg, Germany; 2 United Nations Children's Fund (UNICEF), West and Central Africa Regional Office, Dakar, Senegal; 3 UNICEF Area Representative for Gabon and São Tomé and Príncipe and to the ECCAS, Libreville, Gabon; 4 Department of Economics and Centre for Modern Indian Studies, University of Göttingen, Göttingen, Germany

## Abstract

**Objective:**

Early marriage and childbearing have substantial detrimental effects on both, the affected girls and women at the micro level, as well as entire economies on the macro level. West and Central African countries have some of the highest prevalence rates of early marriage and maternity worldwide. This work attempts to quantify the long-term economic, societal, and fertility effects of marriage and pregnancy in early and late adolescence in West and Central Africa.

**Methods:**

We used pooled cross-sectional data collected between 1986 and 2017 in 21 West and Central African countries within the DHS and MICS programs to estimate the associations of marriage and maternity during early (10-14) and late (15-19) adolescence retrospectively on wealth accumulation, educational attainment, as well as the woman’s lifetime fertility.

**Results:**

Descriptively, women who married or gave birth as young or very young adolescents are overrepresented among the poorest and least educated quintiles of the adult population and underrepresented among the richest and most educated. These gradients were confirmed within a regression analysis which additionally controlled for current age of the woman and PSU fixed effects. Marrying in early/late adolescence was associated with a 12%/6% higher likelihood of being in the poorest wealth quintile in later life and 29%/20% increased likelihood of not completing primary education, as compared to women who married as adults. Maternity in early/late adolescence was associated with a 7%/4% higher likelihood of belonging to the poorest quintile and 17%/10% higher likelihood of being uneducated. Moreover, women who married/gave birth during early or late adolescence, on average, have 2.2/2.3 or 1.4/1.5 more children than those who have married/become mothers as adults.

**Conclusions:**

Our findings suggest that the dire consequences of early marriage and maternity hit youngest girls the hardest – both immediately and long-term. Hence, it is not only worthwhile to prevent adolescent marriage and pregnancy in general, but also specifically target very young girls below age 15 to attempt to at least delay such far-reaching demographic life events.

Child marriage (any legal or customary union before age 18) is prohibited by international law in most countries of the world and various international treaties, conventions, or resolutions address this issue (eg, Sustainable Development Goals, the Convention on the Rights of the Child, the Convention on the Elimination of All forms of Discrimination against Women, the Universal Declaration of Human Rights). Yet in the year 2017, globally, at least 100 million girls were estimated to not be effectively legally protected against this horrendous injustice [1]. This means that a country does not define legal age of marriage at all or the legal age of marriage is below 18 years of age for both or one spouse (usually the wife) on the one hand, or that while legal age of marriage is 18 or above, marriage at younger ages is still possible through parental or judicial consent or other exceptions. In addition, many marriages involving minors are officiated illegally. While boys, too, can be exposed to child marriage, the practice is more common and more damaging for young girls, who suffer societal, economic, and health consequences. These ‘child brides’ usually experience ‘overlapping vulnerabilities’, as they are typically young, poor, and undereducated [2].

When examining the driving forces behind the continuation of early marriage, it is important to look at both ‘demand’ and ‘supply’ side of the equation [3]: on the one hand, men might perceive younger brides as particularly attractive because they are less assertive and hence easier to integrate into an existing household with its hierarchical structure, or because they have a higher expected lifespan fertility, or can perform more strenuous household chores. On the other hand, parents (or other guardians) of young girls may want to minimize their expenditures or seek better financial and physical protection (for instance from sexual advances from men) for their daughters. In addition to that, cultures in which financial transactions – bride price/wealth or dowry – accompany marriages, oftentimes have their incentive structures aligned in a way that incentivizes marrying off daughters as young as possible.

Girls who marry as minors are substantially more likely to experience adverse physical and mental health consequences: most prominently through pregnancy and childbirth early in life while their bodies may not yet be fully developed for procreation. Research shows that adolescent mothers are at substantially greater risk of pregnancy and birth complications as well as maternal mortality (compared to adult women) which are, in turn, correlated with elevated health risks for their offspring. Moreover, former child brides are less likely to use modern contraception later in life, exposing them disproportionally to further disadvantages such as short birth spacing intervals, higher total fertility, or sexually transmitted infections (STIs). In addition to these, young girls living with their (older) husbands and in-laws are at increased risk of physical and emotional violence from the families they have married into [4]. What is more, marriage and pregnancy are among some of the most prominent causes for school drop-out among adolescent girls [2,5]. This translates not only to insufficient literacy and numeracy skills but also reduced employability of young women.

These individual predicaments add up to adverse macro-level effects: Chaaban and Cunningham estimate lifetime opportunity cost related to adolescent pregnancy (measured by foregone lifetime income) [6]. They arrive at estimates of astonishing 30% of annual GDP in Uganda, 27% in Malawi, and 26% of GDP in Nigeria (for comparison, China only foregoes about 1% of its annual GDP due to premature childbearing). Globally, the incidence of child marriage is estimated at 40.34% for the most recent birth cohort (1985-1989) [7]. Of all regions, particularly South Asia (SA) and Sub-Saharan Africa (SSA) are notorious for their persistently high child marriage rates with 45.43% and 38.52%, respectively, with Sub-Saharan-Africa having particularly unsustainable reduction rates in child marriage, leaving the region at high risk of not achieving the SDG of ending child marriage by 2030. Moreover, the authors also estimate a Foster-Greer-Thorbecke-type index called “marriage gap” which weights child marriage incidence with data on how early the girls marry and they arrive at 5.95% globally, 6.09% in SSA and 7.02% in SA (for a discussion of the index see [8]).

Within SSA, West and Central Africa have the highest child marriage incidence of 49% and 40%, respectively [9]. These geographic areas are therefore particularly interesting for a further investigation of the long-term economic and societal as well as fertility consequences of early marriage and pregnancy.

With the present paper we aim to contribute to further understanding of the consequences of early marriage and child bearing by comparing wealth, education and total fertility of adult women (defined as age 20 and above) who married or gave birth as adolescents with that of adult women who married or gave birth later in life.

## METHODS

### Data sources

We pooled all Demographic and Health Surveys (DHS) and Multi Indicator Cluster Surveys (MICS) for West and Central African countries that were conducted between 1986 and 2017. The sample includes women that at the time of the survey were adults (aged 20 years or above) and had been married at least once.

Countries included in the analyses are Benin, Burkina Faso, Cameroon, Central African Republic, Chad, Congo, Congo, Dem. Rep., Cote d'Ivoire, Gabon, Gambia, Ghana, Guinea, Guinea Bissau, Liberia, Mali, Mauritania, Niger, Nigeria, Senegal, Sierra Leone, and Togo. Data used for descriptive analyses stems from the most recent survey per country and has been collected between 1994 and 2017, with only one DHS survey (Central African Republic) stemming from the last millennium and the remaining 20 country data sets from years 2000-2017. The final data set contains roughly 1 285 000 observations, with maternity information available for almost all of the observations (1,283,682) and marriage information for 985 347 of them.

### Outcomes

Outcome variables include wealth quintiles which are measured by an asset index, educational attainment with the categories “less than primary education”, “primary or incomplete secondary education” and “secondary education and above”, and the total number of children ever born. In the descriptive analyses all wealth and education categories are shown. In the regression analyses the outcomes are indicator variables for poorest wealth quintile and the lowest education category. For the children ever born the variable is the simple count of children.

### Exposure

Exposure variables are indicator variables for different ages at first marriage and maternity respectively. We follow the UN classification of young adolescents (10-14 years), older adolescents (15-19 years) (also referred to as “early adolescence” and “late adolescence”) and adults (20 years and above). There were a small number of observations that gave birth at age below 10 years, which were considered mostly implausible and thus have been excluded from the analysis.

### Statistical analysis

Descriptive statistics were calculated using survey weights for individual surveys. In the pooled regional sample each country was weighted with its population share to account for regional composition. Regressions for the outcomes “poorest wealth quintile” and “less than primary education” were linear probability models with primary sampling unit (PSU) fixed effects to control for characteristics of the local environment where women live. Moreover, PSU fixed effects implicitly control for year of the survey, as PSUs are survey specific. Linear probability models are the preferred specification in regressions with a larger number of fixed effects, because probit and logit models would possibly be inconsistent. Regressions for the outcome ‘total number of children ever born’ were simple linear models also controlling for primary sampling unit fixed effects. All analyses as well as sample generation have been performed using STATA 14 statistical software package (StataCorp, College Station, TX, USA).

## RESULTS

**Figure 1:**
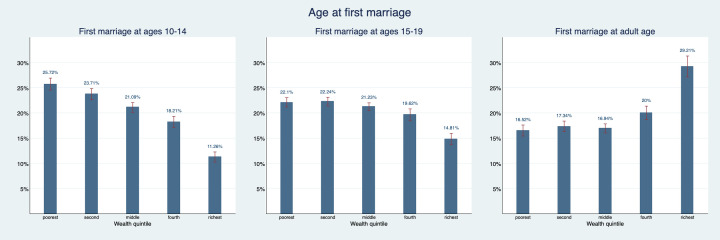
Asset index quintile distribution by age of first marriage. Figure provides point estimates (blue bar) and their corresponding 95% confidence intervals (red bracket).

**Figure 1** shows how women who married as young adolescent, older adolescent or adult are distributed across the different wealth quintiles. We find women who married as young adolescents overrepresented in the poorest wealth quintile and underrepresented in the richest wealth quintile of the population: 25.7% (95% confidence interval (CI) = 24.5%-26.9%) belong to the poorest quintile and only 11.3% (95% CI = 10.3%-12.3%) belong to the richest quintile. Women who married as older adolescents are less likely to belong to the richest quintile with a share of 14.8% (95% CI = 13.7%-16.0%). Their share in the first three quintiles is slightly elevated. In contrast, women who married as adults are overrepresented in the richest quintile and underrepresented in the poorest quintile of the population: 29% (95% CI = 27.2%-31.3%) belong to the richest quintile and 16.5% (95% CI = 15.4%-17.6%) belong to the poorest quintile.

**Figure 2:**
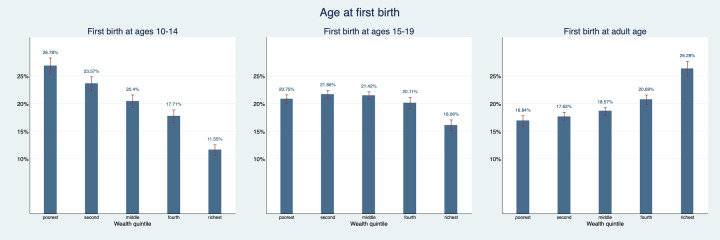
Asset index quintile distribution by maternal age at first birth. Figure provides point estimates (blue bar) and their corresponding 95% confidence intervals (red bracket).

**Figure 2** explores the same relationship but for age at first birth instead of marriage. Patterns are also quite similar: 26.8% (95% CI = 25.3%-28.2%) of women who gave birth as young adolescent are part of the poorest wealth quintile and only 11.6% (95% CI = 10.6%-12.5%) are part of the richest wealth quintile. For women who gave birth as older adolescents the share in the richest wealth quintile is only 16.1% (95% CI = 15.1%-17.1%). Women who gave first birth as adults are overrepresented in the richest quintile with 26.3% (95% CI = 24.9%-27.6%) and underrepresented in the poorest quintile with just 16.8% (95% CI = 15.9%-17.8%).

**Figure 3:**
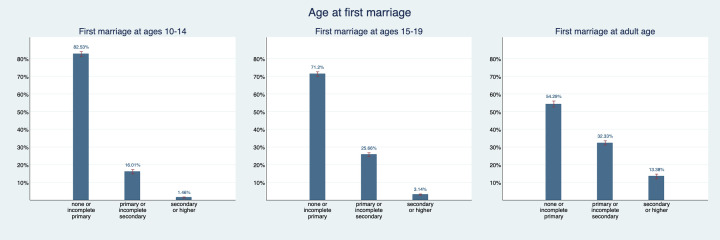
Educational attainment distribution by age of first marriage. Figure provides point estimates (blue bar) and their corresponding 95% confidence intervals (red bracket).

**Figure 4:**
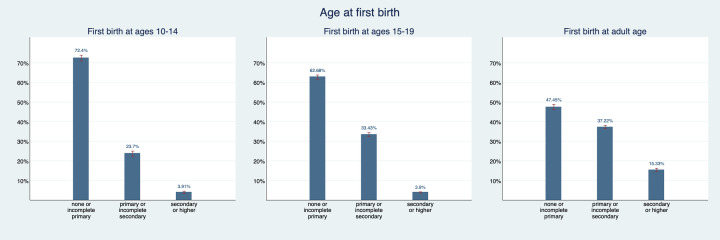
Educational attainment distribution by maternal age at first birth. Figure provides point estimates (blue bar) and their corresponding 95% confidence intervals (red bracket).

**Figure 3** and **Figure 4** show patterns in the education distribution. 82.5% (95% CI = 81.2%-83.8%) of women who married as young adolescents and 71.2% (95% CI = 69.9%-72.5%) of women who gave birth as young adolescents have less than primary education and only 1.5 (95% CI = 1.1%-1.8%) and 3.9% (95% CI = 3.6%-4.2%) respectively have secondary or higher education. For women who gave first birth as older adolescents, the share with less than primary education is about 10 percentage points lower and the share with primary educations is almost 10 percentage points higher than for women who gave first birth as young adolescents. The share with secondary education is very similar in both groups. For women who were first married or gave first birth as adults, this looks quite different: 13.4% (95% CI = 12.1%-14.7%) of women who were first married as adults have secondary education and 54.3% (95% CI = 52.6%-56.0%) have less than primary education. 15.3% (95% CI = 14.4%-16.3%) of women who became mothers as adults have secondary education and 47.5% (95% CI = 46.1%-48.8%) have less than primary education. Hence, we can say that we observe a gradient in educational attainment and economic prosperity based on age at which a woman married and had her first child.

In **Table 1** and **Table 2** we investigate these patterns in a regression analysis controlling for primary sampling unit fixed effects and current age of the women. Age is typically correlated with wealth, education, and total number of children ever born. Controlling for age ensures that we compare women of equal age to each other, who only differ in the timing of first marriage and first childbearing. After adjusting for a dummy for first birth/marriage as each, young or old adolescent, current age, and primary sampling unit fixed effects (PSU FE), adult women who married as young adolescents are still 11.6 percentage points more likely to belong to the poorest wealth quintile than women who first married as adults. Women who married as older adolescents are still 6.4 percentage points more likely to be part of the poorest wealth quintile in comparison to women who married as adults. For first birth these likelihoods are elevated by 7.0 (young adolescent) and 4.0 (older adolescent) percentage points respectively.

**Table 1:** Regressions of select socio-economic characteristics on adolescent marriage

	(1)	(2)	(3)	(4)	(5)	(6)
	**Poorest wealth quintile**	**Poorest wealth quintile with PSU FE**	**Below primary education**	**Below primary education with PSU FE**	**Total children ever born**	**Total children ever born with PSU FE**
Married at age 10-14	0.126‡ (0.003)	0.116‡ (0.003)	0.311‡ (0.003)	0.290‡ (0.003)	2.184‡ (0.010)	2.171‡ (0.010)
Married at age 15-19	0.072‡ (0.002)	0.064‡ (0.002)	0.216‡ (0.002)	0.200‡ (0.002)	1.462‡ (0.007)	1.443‡ (0.007)
Current age	0.000† (0.000)	0.000‡ (0.000)	0.003‡ (0.000)	0.004‡ (0.000)	0.225‡ (0.000)	0.225‡ (0.000)
Constant	0.165‡ (0.004)	0.169‡ (0.003)	0.468‡ (0.004)	0.475‡ (0.003)	-4.313‡ (0.014)	-4.305‡ (0.016)
Observations	515 909	515 909	588 929	588 929	623 694	623 694

PSU FE – primary sampling unit fixed effects

*Standard errors in parentheses.

†*P* < 0.05.

‡*P* < 0.001.

**Table 2:** Regressions of select socio-economic characteristics on adolescent maternity

	(1)	(2)	(3)	(4)	(5)	(6)
	**Poorest wealth quintile**	**Poorest wealth quintile with PSU FE**	**Below primary education**	**Below primary education with PSU FE**	**Total children ever born**	**Total children ever born with PSU FE**
First birth at age 10-14	0.080‡ (0.003)	0.069‡ (0.003)	0.188‡ (0.003)	0.171‡ (0.002)	2.342‡ (0.012)	2.307‡ (0.012)
First birth at age 15-19	0.048‡ (0.001)	0.040‡ (0.001)	0.117‡ (0.002)	0.102‡ (0.001)	1.486‡ (0.006)	1.463‡ (0.006)
Current age	0.000 (0.000)	0.000† (0.000)	0.004‡ (0.000)	0.005‡ (0.000)	0.221‡ (0.000)	0.222‡ (0.000)
Constant	0.191‡ (0.004)	0.191‡ (0.003)	0.507‡ (0.004)	0.508‡ (0.003)	-3.852‡ (0.012)	-3.853‡ (0.015)
Observations	554 182	554 182	611 679	611 679	680 079	680 079

PSU FE – primary sampling unit fixed effects

*Standard errors in parentheses.

†*P* < 0.01.

‡*P* < 0.001.

Similarly, for education, women who first married as young adolescents are 29 percentage points more likely to have less than primary education than women who first married as adults. Women who first married as older adolescents are 20 percentage points more likely than women who first married as adults to have less than primary education. For age at first birth these differences are 17 and 10 percentage points respectively.

Total fertility is substantially higher for women who married or gave birth for the first time early. Women who married as young adolescents, on average, have 2.2 more children within their lifetime (up to the day they are surveyed) than women who first married as adults. Women who married as older adolescents, on average, have 1.4 more children than women who first married as adults. For age at first birth total fertility is increased by 2.3 and 1.5 children respectively.

**Figure 5:**
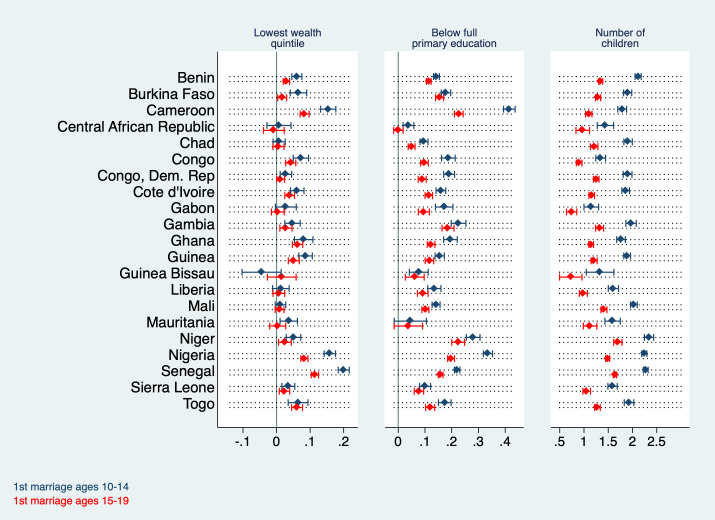
Coefficients from regressions of various outcome variables (belonging to the lowest wealth quintile/having less than primary education / number of children) on the regressors ‘young adolescent marriage’ and ‘older adolescent marriage’ by country (controlled for current age and primary sampling unit fixed effects, PSU FE). Regression coefficients are shown by country (diamond) with their corresponding 95% confidence intervals (bracket).

**Figure 6:**
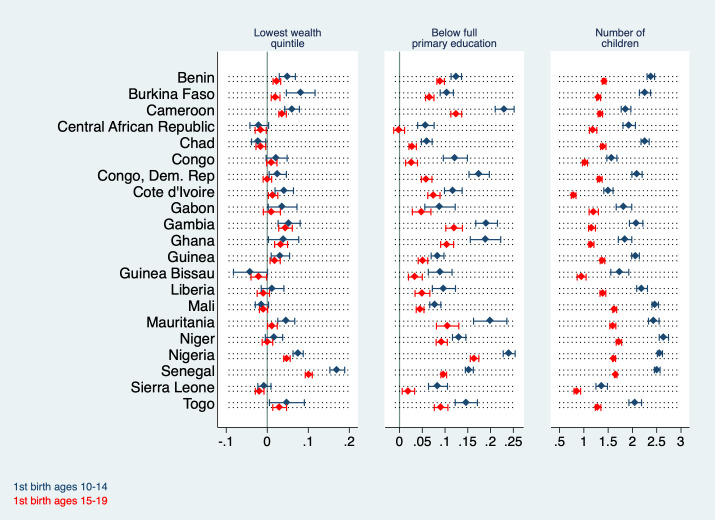
Coefficients from regressions of various outcome variables (belonging to the lowest wealth quintile / having less than primary education / number of children) on the regressors ‘first birth as young adolescent’ and ‘first birth as older adolescent’ by country (controlled for current age and primary sampling unit fixed effects, PSU FE). Regression coefficients are shown by country (diamond) with their corresponding 95% confidence intervals (bracket).

**Figure 5** and **Figure 6** display regression coefficients for models 2, 4, and 6 in **Table 1** and **Table 2** by country. It is notable that regressing wealth on adolescent marriage yields particularly high coefficients in Cameroon, Nigeria, and Senegal where being married in young adolescence is associated with a roughly 20 percentage points higher probability of belonging to the poorest quintile. At the same time, the coefficients are comparatively low (below 10 percentage points) and statistically insignificant in Central African Republic, Chad, Gabon, Guinea Bissau, Liberia, and Mali. The pattern is similar for adolescent maternity; here the coefficients are statistically insignificant for Central African Republic, Chad, Congo, Guinea Bissau, Liberia, Mali, Niger and Sierra Leone. For all of these countries with insignificant coefficients except Congo and Liberia this association is negative. Chad is the only country with a negative significant association. The association between adolescent marriage and maternity with education is statistically significant in all countries except in Central African Republic (both marriage and maternity insignificant for older adolescents) and Mauritania (younger and older adolescent marriage insignificant). In Cameroon, a woman who married in young adolescence has a 42 percentage points higher probability of being uneducated (95% CI = 0.39-0.44) than one who married later in life. In Nigeria, this excess probability amounts to 34 percentage points (95% CI = 0.32-0.35) and in Niger to 28 percentage points (95% CI = 0.25-0.30).

Early marriage and maternity are associated with a statistically significant increase in fertility in all countries, with the corresponding coefficients ranging from 1.15 in Gabon (young adolescent marriage) to 2.6 in Niger (young adolescent maternity). This means that women in Niger who gave birth between ages 10 and 14 have, on average, 2.6 more children than women who became mothers at a later point in life. The association between older adolescent marriage and maternity with total fertility is slightly weaker than for young adolescent marriage and maternity, however nonzero and statistically significant across all countries.

## DISCUSSION

We have explored select socio-economic characteristics (wealth status and educational attainment) as well as total fertility of women who married or gave birth as adolescents several years later in their lives, as adults. We find that women who married or gave birth as adolescents, and particularly as young adolescents aged 10-14, are disadvantaged in socio-economic terms as adults. What is more, their total fertility is substantially higher – over the course of their lives (we can only account for their lifetime up to the survey date), these women, on average, have over two children more than women who married or became mothers as adults.

We find that these effects are detrimental and ‘sticky’ in the sense that they persist over the woman’s lifespan. Because the negative impact on girls’ lives is even greater with larger deviations from the desired legal age of marriage (“young adolescents” vs “older adolescents” distinction in our work) we confirm that, in line with Nguyen and Wodon’s work [8], how much too early young girls marry is indeed an important piece of information to consider besides the general child marriage incidence rates.

We have previously learnt through our research [10] that women who marry or give birth as adolescents are socio-economically disadvantaged even prior to being married. Although our outcome variables are measured several years after the exposure, we cannot claim that we identify causal effects. Ideally, we would like to control for initial socio-economic conditions, but unfortunately, these are not measured retrospectively in the DHS and MICS surveys. We therefore have to interpret all findings as descriptive.

One interesting area of further study is the school drop-out point and its justification; whereas a substantial body of literature places marriage and childbirth into direct context with premature school leaving, there is evidence from non-standard DHS questionnaires in five Francophone SSA countries – Burkina Faso, Guinea, Côte d’Ivoire, Cameroon, and Togo – that premature school leaving is mostly explained by factors other than marriage and childbirth, and that between these two, marriage incurs a higher drop-out risk than childbirth [11].

## CONCLUSION

We investigated long-term effects of adolescent marriage and childbearing in West and Central Africa and found them to be both detrimental and sticky in the way they affect women’s lives over their entire lifespan. We furthermore found that very young girls are even more vulnerable to negative consequences of child marriage. Hence it appears desirable to certainly fight child marriage in all of its forms, but also particularly focus on delaying it as long as possible where a complete annihilation might not be feasible. With West and Central Africa being highly heterogeneous in its customs and cultural settings the path to eradication of child marriage will have to be context-specific. However, one common trait that appears to distinguish this region from others (most prominently from South Asia), is that in West and Central Africa, sexual activity among unmarried adolescents is common in most settings while at the same time, girls generally value education and prefer to stay in school over early marriage or childbirth [5]. Hence an even greater emphasis needs to be placed on information, empowerment, and provision campaigns aimed at young girls (and boys) to improve access to and knowledge about modern contraceptive methods. The importance of this approach is corroborated by a systematic review from Sub-Saharan Africa that identifies cost of contraceptives and lack of adolescent-friendly health service provision as the major impediment to adequate reproductive health in the region [12]. Simultaneously, the educational system needs to be modified in a way which stimulates particularly secondary education for girls, as this is where the largest bottleneck persists. This would entail boosting timely entry into primary school for all girls, as under current circumstances, a substantial share of girls lag behind in their education which is identified as a significant driver behind low secondary continuation rates [13]. Another important aspect is to allow for effortless re-entry into the educational system for young women who have temporarily interrupted their education due to demographic life events such as marriage or childbirth. Determining how to achieve these goals is one of the major challenges of evidence-based education research. Indeed, evidence available to date appears to be rather mixed and, again, highly context-specific, as seemingly identical interventions (eg, school uniform provision) can have vastly different effects across the African continent [14]. In addition to the above mentioned endeavors, countries should direct more effort into enforcing minimum legal age laws where such laws already exist and establishing them otherwise, as consistent laws against the practice of child marriage are empirically shown to be associated with lower prevalence of child marriage [15,16].

## References

[1] Wodon Q, Tavares P, Fiala O, Le Nestour A, Wise L. Child Marriage Laws and their Limitations. End Child Marriage Notes Ser. Washington DC: World Bank; 2017.

[2] Parsons J Edmeades J Kes A Petroni S Sexton M Wodon Q Economic impacts of child marriage: a review of the literature. Rev Faith Int Aff. 2015;13:12-22. 10.1080/15570274.2015.1075757

[3] Jensen R Thornton R Early female marriage in the developing world. Gend Dev. 2003;11:9-19. 10.1080/741954311

[4] Raj A When the mother is a child: the impact of child marriage on the health and human rights of girls. Arch Dis Child. 2010;95:931-5. 10.1136/adc.2009.178707 20930011

[5] Petroni S Steinhaus M Fenn NS Stoebenau K Gregowski A New findings on child marriage in sub-Saharan Africa. Ann Glob Health. 2017;83:781-90. 10.1016/j.aogh.2017.09.001 29248095

[6] Chaaban J, Cunningham W. Measuring the economic gain of investing in girls: the girl effect dividend. Washington DC: The World Bank; 2011.

[7] Nguyen MC Wodon Q Global and Regional Trends in Child Marriage. Rev Faith Int Aff. 2015;13:6-11. 10.1080/15570274.2015.1075756

[8] Nguyen MC Wodon Q Measuring child marriage. Econ Bull. 2012;32:398-411.

[9] Walker J-A Early marriage in Africa–trends, harmful effects and interventions. Afr J Reprod Health. 2012;16:231-40. 22916555

[10] Sagalova V Garcia J Kapeu AS Zagre NM Vollmer S Socio-economic predictors of adolescent marriage and maternity in West and Central Africa between 1986 and 2017. J Glob Health. 10.7189/jogh.11.13002 PMC8397280 34484709

[11] Lloyd CB Mensch BS Marriage and childbirth as factors in dropping out from school: an analysis of DHS data from sub-Saharan. Popul Stud (Camb). 2008;62:1-13. 10.1080/00324720701810840 18278669

[12] Yakubu I Salisu WJ Determinants of adolescent pregnancy in sub-Saharan Africa: a systematic review. Reprod Health. 2018;15:15. 10.1186/s12978-018-0460-4 29374479 PMC5787272

[13] World Health Organization. Social determinants of sexual and reproductive health: informing future research and programme implementation. Geneva: WHO; 2010.

[14] Kalamar AM Lee-Rife S Hindin MJ Interventions to Prevent Child Marriage Among Young People in Low- and Middle-Income Countries: A Systematic Review of the Published and Gray Literature. J Adolesc Health. 2016;59:S16-21. 10.1016/j.jadohealth.2016.06.015 27562449

[15] Maswikwa B Richter L Kaufman J Nandi A Minimum Marriage Age Laws and the Prevalence Of Child Marriage and Adolescent Birth: Evidence from Sub-Saharan Africa. Int Perspect Sex Reprod Health. 2015;41:58-68. 10.1363/4105815 26308258

[16] Sagalova V Garcia J Bärnighausen T Ntambi J Sodjinou R Zagre NM Vollmer S Levels and trends of adolescent marriage and maternity in West and Central Africa, 1986-2017. J Glob Health. 2021;11:13001. 10.7189/jogh.11.13001 PMC8397283 34484708

